# Systematic comparison of four point-of-care methods versus the reference laboratory measurement of hemoglobin in the surgical ICU setting: a cross-sectional method comparison study

**DOI:** 10.1186/s12871-020-01008-8

**Published:** 2020-04-22

**Authors:** Arpa Chutipongtanate, Churairat Yasaeng, Tanit Virankabutra, Somchai Chutipongtanate

**Affiliations:** 1 Department of Anesthesiology, Faculty of Medicine Ramathibodi Hospital, Mahidol University, Bangkok, Thailand; 2 Pediatric Translational Research Unit, Department of Pediatrics, Faculty of Medicine Ramathibodi Hospital, Mahidol University, 270 Rama VI Rd., Ratchathewi, Bangkok, 10400 Thailand; 3 Department of Clinical Epidemiology and Biostatistics, Faculty of Medicine Ramathibodi Hospital, Mahidol University, Bangkok, Thailand

**Keywords:** Agreement, Bias, Correlation, Hemoglobin measurement, Point-of-care testing

## Abstract

**Background:**

Transfusion decision during the perioperative period mostly relies on the point-of-care testing for Hb measurement. This study aimed systematically compared four point-of-care methods with the central laboratory measurement of hemoglobin (LHb) regarding the accuracy, precision, and assay practicality to identify the preferred point-of-care method during the perioperative period.

**Methods:**

This cross-sectional method comparison study was conducted in the surgical intensive care unit at Ramathibodi Hospital, Thailand, from September 2015 to July 2016. Four point-of-care methods, i.e., capillary hematocrit (HctCap), HemoCue Hb201+, iSTAT with CG8+ cartridge, and SpHb from Radical-7 pulse co-oximeter were carried out when LHb was ordered. Pearson correlation and Bland-Altman analyses were performed to assess the accuracy and precision, while the workload, turnaround time, and the unit cost were evaluated for the method practicality.

**Results:**

Thirty-five patients were enrolled, corresponding to 48 blood specimens for analyses, resulting in the measured hemoglobin of 11.2 ± 1.9 g/dL by LHb. Ranking by correlation (*r*), mean difference (bias) and 95% limit of agreement (LOA) showed the point-of-care methods from the greater to the less performance as followed, iSTAT-LHb pair (*r* = 0.941; bias 0.15 (95% LOA; − 1.41, 1.12) g/dL), HemoCue-LHb pair (*r* = 0.922; bias − 0.18 (95% LOA; − 1.63, 1.28) g/dL), SpHb-LHb pair (*r* = 0.670; bias 0.13 (95% LOA; − 3.12, 3.39) g/dL) and HctCap-LHb pair (*r* = 0.905; bias 0.46 (95% LOA; − 1.16, 2.08) g/dL). Considering the practicality, all point-of-care methods had less workload and turnaround time than LHb, but only HemoCue and HctCap had lower unit cost.

**Conclusion:**

This study identified HemoCue as the suitable point-of-care method for the sole purpose of Hb measurement in the surgical ICU setting, while iSTAT should be considered when additional data is needed.

## Introduction

Acute anemia due to bleeding is a significant complication that causes morbidity and mortality in patients during the perioperative period. Severe anemia leads to inadequate oxygen delivery to the tissues. The decision to treat anemia rely on both clinical signs of inadequate oxygen delivery and laboratory parameter [1–4]. Hemoglobin (Hb) concentration is the mainstay parameter to evaluate acute anemia in both operating room and intensive care unit (ICU). Although Hb measurement by the central laboratory (LHb) is the gold standard method, its official report is usually delayed due to time-consuming processes such as specimen transport and report generation. Thus, transfusion decision during the perioperative period mostly relies on point-of-care testing (POCT) for Hb measurement.

POCT for Hb measurement can be classified as invasive hemoglobin measurement, i.e., hematocrit capillary tube centrifugation (HctCap), HemoCue, and iSTAT), and non-invasive hemoglobin monitoring (SpHb) such as Radical-7 Pulse CO-Oximeter [5–9]. HctCap is the conventional method to measure hematocrit (Hct) level by using a centrifugal force to sediment red blood cells (RBC) expressed as the percentage of the sediment RBC to the whole blood volume measured. Hb is then estimated from Hct divided by three. HemoCue is POCT that provides immediate hemoglobin values base upon a modified azide methemoglobin reaction and dual wavelengths (570 nm and 880 nm) detection for compensation of turbidity. HemoCue uses a minimal blood volume (10 μL) for an analysis. iSTAT is another POCT which measures Hct (and then calculate for Hb level) based on microfluidic conductometry. This method needs a few drops of the blood sample to fill into a cartridge, which is then inserted into the iSTAT handheld to measure Hb concentration. Radical-7 Pulse CO-Oximeter can be applied for SpHb measurement based on spectrophotometry using multi-wavelength light absorption.

To select a suitable POCT for Hb measurement during the perioperative period, the method accuracy and precision have to be compared with the reference Hb measurement from the central laboratory. Also, the practicality of POCT, including the workload, turnaround time, and unit cost, should be taken into account. This study, therefore, aimed to systematically compare the accuracy, precision, and practicality of four POCT, including HctCap, iSTAT, HemoCue, and SpHb, against the reference LHb in the surgical ICU setting. The findings of this study may also apply to select the suitable POCT for Hb measurement in other contexts and settings.

## Methods

### Study design

This cross-sectional method comparison study was conducted at the surgical ICU, Ramathibodi Hospital from September 2015 to July 2016. The eligible criteria including; patients age ≥ 18 years old who were admitted to the surgical ICU, had arterial line placement intraoperatively, and had LHb ordered within the perioperative period. The patient who was unable to use pulse oximetry device (i.e., extremities amputation, severe burn) or received vasopressors was excluded from the study. The informed consent was obtained directly from participating patients or from a legally authorized representative when the patient was not able to provide consent. This study protocol was approved by the Ethical Clearance Committee on Human Right Related to Research involving Human Subjects, Faculty of Medicine Ramathibodi Hospital, Mahidol University (ID 06–58-24).

### Hb measurement

For eligible patients, blood collection for Hb measurement was performed at the same time as the LHb request within 24-h postoperative, for example, suspicious postoperative anemia or acute blood loss in the ICU. Three milliliters of blood was gently drawn through the radial 20-gauge arterial catheter into a 6-ml EDTA tube. The reference LHb was performed as part of the complete blood count using the ADVIA 2120 hematology system. Note that there was no blood specimen with hemolysis reported from the reference LHb. The POCT methods were run in parallel using the same EDTA blood specimen. HctCap was performed by a microhematocrit centrifuge, while HemoCue (HemoCue® Hb-201+; HemoCue AB, Ängelholm, Sweden) and iSTAT (iSTAT-1 with CG8+ cartridges; I-STAT Corp., Princeton, NJ) were performed as the manufacturer instructions. At the same time of blood collection, Radical-7 Pulse CO-Oximeter using the R2–25 sensor system was used to measure SpHb level on the contralateral extremity to the arterial line insertion. The LHb is externally calibrated annually and internally calibrated using the quality control reagent two times daily. HemoCue and SpHb are factory calibrated and need no further calibration by the end-user. iSTAT has been externally calibrated every 6 months (at approximately 3 months before and 3 months after the study initiation). In addition, the iSTAT calibration has been performed by the ICU staff using the liquid quality control weekly, and the electronic stimulator test has been carried out at 4 am daily or at the first analysis of the day.

### Data collection

Demographic and clinical data including age, gender, American Society of Anesthesiologists physical classification (ASA class), preoperative Hb, estimated blood loss, intraoperative transfusion, and types of surgery were collected by the medical chart review. The measured Hb levels from different methods were obtained as aforementioned. The step of the procedure was adopted as representative of the procedure workload. The step of procedure for each technique was described as following; LHb, 6 steps (draw blood, label the sample, transport the sample, perform lab analysis, generate the report, read the result); HctCap, 4 steps (draw blood, fill blood into a capillary tube, centrifugation, read the result); HemoCue, 4 steps (draw blood, fill blood into a microcuvette, insert a microcuvette into the machine, read the result); iSTAT, 4 steps (draw blood, fill blood into a cuvette, insert a cuvette into the machine, read the result); SpHb, 2 steps (place the sensor, read the result). Turnaround time was defined by the estimated time required from the start of the procedure until the result obtained. Turnaround time of the LHb also depended on the reported time as recorded in the Electronic Medical Record. The unit cost (based the exchange rate on May 22, 2019) was estimated by consumable supplies (i.e., capillary tube, cuvette/microcuvette, or sensors) but not included the cost of the instrument or reusable device.

### Sample size calculation and statistical analysis

The sample size was calculated by power analysis for correlation test using *pwr* package. By the assumption that the correlation (*r*) of the measured Hb between LHb and POCT was not lower than 0.6 (the moderate correlation), the sample size of 34 was required to meet the significant level (alpha) of 0.01 and the power of 90%.

Statistical analysis was performed by Excel and R programs. Categorical data are reported as numbers and percentages. Quantitative data are reported as mean ± SD, or median [IQR] as appropriate. Quantile–Quantile plots of the differences were performed to visually validate the data normality as shown in Supplementary Figure 1. Correlation (*r*) between the reference LHb and Hb values of the POCT methods was performed by Pearson correlation. Agreement of Hb values between the reference LHb and the POCT methods was determined by the Bland-Altman plot. Differences between each pair of measurements (the POCT method - LHb) were plotted on the vertical axis against the averages of the pair (the POCT method + LHb)/2 on the horizontal axis [10]. The Bland-Altman analysis determines the mean of differences (or bias) as a measure of accuracy [10, 11], in which small bias indicated high accuracy of the measurement. The 95% Limit of Agreement (LOA) was defined by ±1.96 SD of the bias [10, 11]. The narrow 95% LOA means high precision of measurement [10, 11]. The acceptable level of bias between the POCT method and LHb was ±4% of the target as defined by Clinical Laboratory Improvement Amendments (CLIA, 2019) [12], and the acceptable 95% LOA was expected to fall within a range of 3 (±1.5 from the mean of differences) as defined by clinically relevant changes of Hb levels. *P*-value < 0.05 was considered statistically significant.

## Results

This cross-sectional method comparison study was conducted to compare five methods of Hb measurement, including 4 POCT and 1 LHb, to identify the most preferred POCT for Hb measurements based on accuracy, precision, and assay practicality. A total of 35 postoperative patients admitted to the surgical ICU were included. Of these, 28 patients had one LHb ordered, and 13 patients had two LHb ordered within 24-h postoperative period, resulting in a total of 48 blood specimens for further analyses. The POCT for Hb measurements (HctCap, HemoCue, iSTAT, SpHb) were simultaneously performed when LHb was ordered, and none were performed while the subject was on vasopressors or received blood transfusion. Patient demographic data were summarized in Table 1.

**Table 1:** Baseline characteristics of patients and surgical procedures

**Characteristics**	*n* = 35 patients
**Age** (year), mean	62.7 ± 17.1
**Male gender,** n (%)	11 (31.4)
**ASA class,** n (%)	
I	2 (5.7)
II	6 (17.1)
III	9 (25.7)
IV	17 (48.6)
V	1 (2.9)
**Preoperative Hb** (g/dL), mean ± SD	11.01 ± 1.92
**Estimated blood loss** (mL), median [IQR]	350 [20, 1350]
**Intraoperative transfusion** (mL), median [IQR]	0 [0, 779]
**Surgical type**, n (%)	
Hepatobiliary surgery	6 (17.1)
Neurosurgery	6 (17.1)
Abdominal surgery	5 (14.2)
Urological surgery	4 (11.4)
Abdominal aortic aneurysm	3 (8.6)
Otolarynx surgery	3 (8.6)
Spinal surgery	3 (8.6)
Obstetrics-Gynecology surgery	1 (2.9)
Debridement	1 (2.9)
Others	3 (8.6)

Abbreviations: *ASA* American Society of Anesthesiologists

**Fig. 1:**
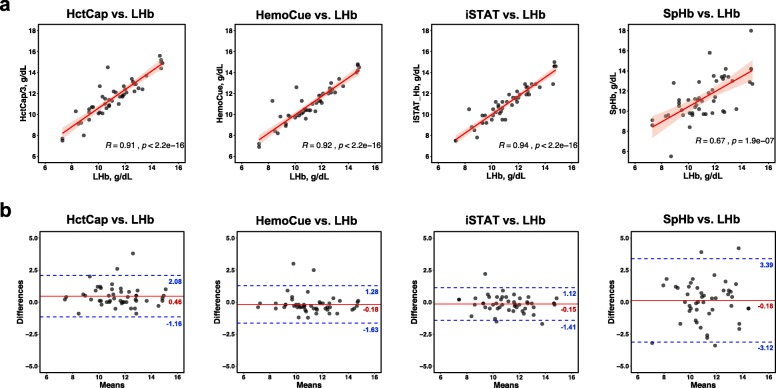
Correlation and agreement between point-of-care testings and the reference central laboratory for hemoglobin measurement. **a** Scatter plot with Pearson correlation analysis. The red color line showed a linear regression curve, where the light-red band represented the 95% confidence interval. **b** Bland-Altman analysis. A horizontal solid line corresponds to the estimated bias, while two horizontal dash lines represent the upper and lower prediction limits, corresponding to the 95% limit of agreement

The scatter plots of paired Hb values and the Bland-Altman plots of the POCT method vs. LHb are shown in Fig. 1, while Table 2 summarizes mean ± SD of the measure Hb, the correlation, agreement and assay performance of analytical methods. Overall, all POCT devices had significantly correlated with LHb (*p* < 1e-6) but at various degrees of the correlation coefficient. The iSTAT-LHb pair (*r* = 0.941), HemoCue-LHb pair (*r* = 0.922) and HctCap-LHb pair (*r* = 0.905) showed excellent correlation, whereas SpHb-LHb pair (*r* = 0.670) had moderate correlation (Fig. 1a and Table 2). This correlation data supported further evaluation of method agreement, including the accuracy and precision, using the Bland-Altman analysis.

**Table 2:** The systematic comparison of five hemoglobin measurements regarding correlation, agreement, and assay practicality (*n* = 48 specimens)

	**Hb (g/dL), mean ± SD**	**Correlation coefficient (*r*)**	**Agreement**				**Practicality**		
			**Mean difference (bias)**	**%Bias from the reference**	**SD**	**95% LOA**	**Step of procedure**^**a**^	**Turnaround time (min)**	**Unit cost (USD)**^**b**^
**LHb (reference)**	11.2 ± 1.9	1.000	–	–	–	–	6	30–60	1.6
**HctCap**	11.7 ± 1.9	0.905	0.46	4.1	0.83	−1.16, 2.08	4	6–10	0.1
**HemoCue**	11.1 ± 1.8	0.922	−0.18	1.6	0.74	−1.63, 1.28	4	1–2	1.3
**iSTAT**	11.1 ± 1.8	0.941	−0.15	1.4	0.65	−1.41, 1.12	4	2–3	8.1
**SpHb**	11.4 ± 2.2	0.670	0.13	1.2	1.66	−3.12, 3.39	2	< 1	65.6

^a^Details in the Materials and Methods section

^b^Based on the exchange rate on May 22, 2019. Note that the unit cost can vary in different settings and countries

Agreement between the POCT device and the reference LHb was evaluated by Bland-Altman analysis, in which the mean of difference (or bias) with the 95% LOA describes the accuracy and precision of the POCT method, respectively. The biases of HctCap, HemoCue, iSTAT and SpHb were 0.46 g/dL, − 0.18 g/dL, − 0.15 g/dL and 0.13 g/dL, respectively (Fig. 1b and Table 2). Since the proficiency testing (CLIA, 2019) for Hb measurement was defined at ±4% of the target [12], our results showed that HctCap (the bias of 4.1% of the mean LHb) had marginally failed to meet the indicated cut-off while HemoCue, iSTAT, and SpHb had the acceptable accuracy (bias of 1.6, 1.4 and 1.2% of the mean LHb, respectively) (Fig. 1b and Table 2). Next, iSTAT (95% LOA of − 1.41, 1.12; the range of 2.53) exhibited higher precision than HemoCue (95% LOA of − 1.63, 1.28; the range of 2.91), HctCap (95% LOA of − 1.16, 2.08; the range of 3.24) and SpHb (95% LOA of − 3.12, 3.39; the range of 6.51), respectively (Fig. 1b and Table 2). HctCap and SpHb had failed to meet the acceptable LOA established a priori, suggesting these methods had a lack of precision. The accuracy and precision of HemoCue and iSTAT were quite comparable (Table 2), which was consistent with the previous study [13], even though iSTAT exhibited slightly better performance than HemoCue.

Assay practicality, including steps of the procedure (as the representative of workload), turnaround time, and the unit cost, were compared among five methods of Hb measurement (Table 2). SpHb had less workload and turnaround time (2 steps; < 1 min) as compared to the reference LHb (5 steps; 30–60 min) and other POCT methods (4 steps; different time of 1–10 min) (Table 2). Nevertheless, the SpHb sensor is costly and thus makes the highest unit cost among five methods evaluated in this study (Table 2). HctCap and HemoCue were cheaper than LHb, whereas iSTAT was 5-time more expensive. It should be noted that iSTAT with CG8+ cartridge not only measures the Hb level but also provides results of blood gas panel, major electrolytes (sodium, potassium, ionized calcium) and glucose, all of which are important for patient management in the critical care setting. Overall, comparing assay practicality of five methods identified HemoCue as a versatile and economical method for Hb measurement.

## Discussion

Point-of-care Hb measuring devices have been extensively studied and compared in terms of accuracy and precision [5–8, 13–15]. Previous studies suggested that HemoCue and iStat could be used interchangeably to measure Hb levels [13], whereas SpHb had lower accuracy and precision than HemoCue [14, 15]. HctCap is widely used in developing countries, including Thailand, and many physicians still rely on this method for guiding transfusion; however, its accuracy and precision have rarely been reported. Most studies compared two to three methods [5–8, 13–15], while the head-to-head comparison of multiple Hb measurements regarding the accuracy, precision in conjunction with assay practicality has never been investigated. Knowing these would guide the selection of POCT for Hb measurement to meet the needs of different contexts and settings.

This study systemically compared five methods of Hb measurement including the reference LHb, and 4 POCT devices, i.e., HctCap, HemoCue, iSTAT, and SpHb to identify the preferred POCT for the surgical ICU setting. Although there was no consensus on what was the best POCT for Hb measurement regarding three comparing parameters (i.e., accuracy, precision, and assay practicality), it was clear that HemoCue and iSTAT were more preferred than HctCap and SpHb in terms of the accuracy and precision. Even though HctCap has an advantage regarding the lowest unit cost, its accuracy failed to meet the proficiency testing (CLIA, 2019) for Hb measurement [12]. SpHb may be suitable to use as an adjunct method for continuous monitoring of Hb changes; nevertheless, our finding did not support a transfusion decision based solely on SpHb due to its lack of precision. The accuracy and precision of HemoCue and iSTAT were very close (Table 2) and may be interchangeable for Hb measurement [13]. Nonetheless, this systematic comparison suggested that HemoCue was more suitable than iSTAT for the sole purpose of intermittent Hb monitoring in the surgical ICU setting due to its versatility and cost-saving, while iSTAT should be more preferred when information of blood gas, electrolytes, or glucose were in need.

This study had limitations. First, several models of HemoCue analyzers (i.e., Hb-201+, Hb-301, and Hb-801), iSTAT cartridges (e.g., CG8+, EC8+, and CHEM8+) and SpHb sensors (i.e., R2–25, R1–25, and DCI SC-360) are applicable for Hb measurement and may have different efficiency and performance. This study only included Hb-201+, CG8+, and R2–25 as representatives of those device models based on the availability in our setting. Second, this study did not address the applicability of the POCT devices on transfusion decision but focused on their comparability to the reference LHb only. Third, this study had a small sample size (*n* = 35 patients), corresponding to 48 specimens and 240 Hb measurements by five methods. Although this sample size was satisfied by power analysis based on the assumption of multiple Hb measurements having at least a moderate correlation (*r* ≥ 0.60, alpha 0.01, power 90%), one should be aware that it was not necessary to be satisfied by the agreement also. Nevertheless, our findings were in line with previous studies regarding the agreement [13–15] with additional advantages from a higher number of methods compared on three domains of assay performance including accuracy, precision, and practicality. Therefore, we believe our findings are useful for the selection of POCT for Hb measurement, particularly in limited-resource settings. Fourth, the measured Hb values were predominantly in the range above the threshold of transfusion decision. Given no obvious trend in bias observed in this study, the associations could be extrapolated to the lower Hb range. Nonetheless, future studies should be designed to assess the agreement across a range of Hb especially at the ends of the spectrum, where a decision has been made for appropriate management.

## Conclusions

Among the POCT devices compared, HemoCue and iSTAT are the preferred POCT for Hb measurement in the surgical ICU setting regarding their comparable accuracy and precision. HemoCue is the method of choice when considering turnaround time and the unit cost, while iSTAT should be used when additional data is needed.

## Supplementary information

**Additional file 1: Table S1.** Raw demographic data of 35 patients included in the study.

**Additional file 2: Table S2.** Raw data of the measured Hb by the reference LHb and four point-of-care devices (*n* = 48 specimens).

**Additional file 3: Figure S1.** Quantile-Quantile (Q-Q) plot was performed to visually evaluate data normality by comparing two probability distributions of theoretical and sample quantiles. Most data points lay close to a linear diagonal line with some points presented within the 95% confidence interval (the grey color band).

## Data Availability

The datasets containing the raw demographic data from 35 patients (Supplementary Table S1) and the measured Hb from different methods (Supplementary Table S2) that support the findings of this study are made available as Supplementary Materials.

## Abbreviations

ASA: American Society of Anesthesiologists
CLIA: Clinical Laboratory Improvement Amendments
Hb: Hemoglobin
HctCap: Capillary hematocrit
ICU: Intensive care unit
LHb: Central laboratory hemoglobin measurement
LOA: Limit of Agreement
POCT: Point-of-care testing
RBC: Red blood cells
SpHb: Non-invasive hemoglobin monitoring

## References

[1] van KAMPEN E, ZIJLSTRA WG (1961). Standardization of hemoglobinometry. II. The hemiglobincyanide method. Clin Chim Acta.

[2] Lienhart A, Auroy Y, Péquignot F, Benhamou D, Warszawski J, Bovet M (2006). Survey of anesthesia-related mortality in France. Anesthesiology..

[3] Gilliss BM, Looney MR, Gropper MA (2011). Reducing noninfectious risks of blood transfusion. Anesthesiology..

[4] Glance LG, Dick AW, Mukamel DB, Fleming FJ, Zollo RA, Wissler R (2011). Association between intraoperative blood transfusion and mortality and morbidity in patients undergoing noncardiac surgery. Anesthesiology..

[5] Giraud B, Frasca D, Debaene B, Mimoz O (2013). Comparison of haemoglobin measurement methods in the operating theatre. Br J Anaesth.

[6] Tsuei BJ, Hanseman DJ, Blakeman MJ, Blakeman TC, Yang SH, Branson RD (2014). Accuracy of non-invasive hemoglobin monitoring in patients at risk for hemorrhage. J Trauma Acute Care Surg.

[7] Lamhaut L, Apriotesei R, Combes X, Lejay M, Carli P, Vivien B (2011). Comparison of the accuracy of noninvasive hemoglobin monitoring by spectrophotometry (SpHb) and HemoCue® with automated laboratory hemoglobin measurement. Anesthesiology..

[8] Miller RD, Ward TA, Shiboski SC, Cohen NH (2011). A comparison of three methods of hemoglobin monitoring in patients undergoing spine surgery. Anesth Analg.

[9] Steinfelder-Visscher J, Teerenstra S, Gunnewiek JM, Weerwind PW (2008). Evaluation of the i-STAT point-of-care analyzer in critically ill adult patients. J Extra Corpor Technol.

[10] Bland JM, Altman DG (1986). Statistical methods for assessing agreement between two methods of clinical measurement. Lancet..

[11] Montenij LJ, Buhre WF, Jansen JR, Kruitwagen CL, de Waal EE (2016). Methodology of method comparison studies evaluating the validity of cardiac output monitors: a stepwise approach and checklist. Br J Anaesth.

[12] U.S. Department of Health and Human Services: Centers for Medicare, Medicaid Services (2019). 42 CFR part 493. Clinical laboratory improvement amendments of 1988 (CLIA) proficiency testing regulations related to Analytes and acceptable performance. Fed Regist.

[13] Kolotiniuk NV, Manecke GR, Pinsky MR, Banks D (2018). Measures of blood hemoglobin and hematocrit during cardiac surgery: comparison of three point-of-care devices. J Cardiothorac Vasc Anesth.

[14] Hiscock R, Kumar D, Simmons SW (2015). Systematic review and meta-analysis of method comparison studies of Masimo pulse co-oximeters (Radical-7™ or Pronto-7™) and HemoCue® absorption spectrometers (B-hemoglobin or 201+) with laboratory haemoglobin estimation. Anaesth Intensive Care.

[15] Skelton VA, Wijayasinghe N, Sharafudeen S, Sange A, Parry NS, Junghans C (2013). Evaluation of point-of-care haemoglobin measuring devices: a comparison of Radical-7™ pulse co-oximetry, HemoCue (®) and laboratory haemoglobin measurements in obstetric patients. Anaesthesia..

