# Supplementary figures and images for: Systematic comparison of four point-of-care methods versus the reference laboratory measurement of hemoglobin in the surgical ICU setting: a cross-sectional method comparison study
Source: BMC Anesthesiol. 2020 Apr 22;20:92. doi: 10.1186/s12871-020-01008-8 (PMC7175548; doi:10.1186/s12871-020-01008-8)

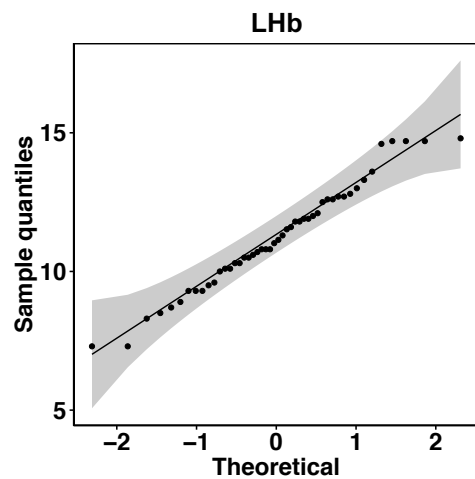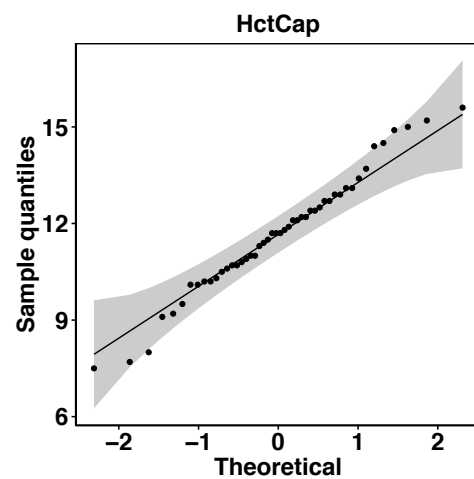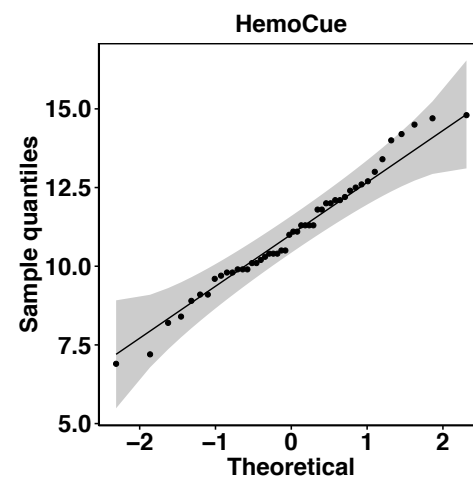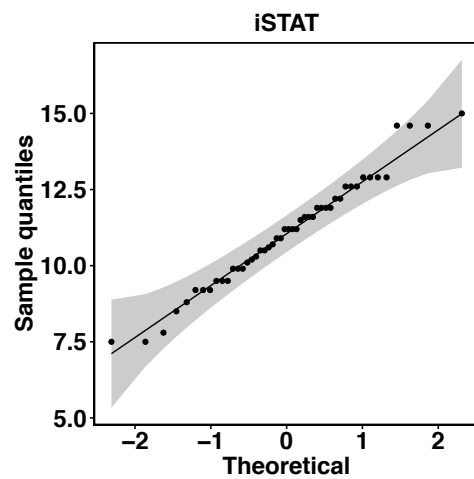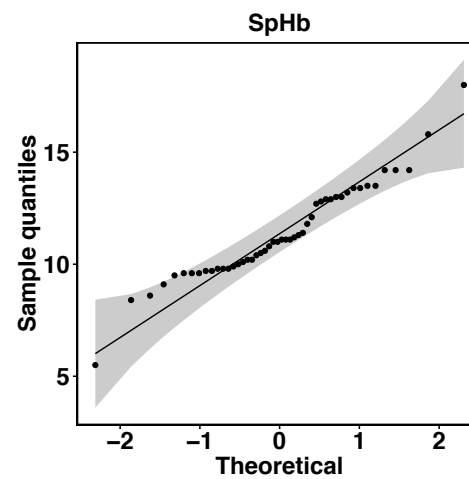

Supplement: Supplementary file 3 — Additional file 3: Figure S1. Quantile-Quantile (Q-Q) plot was performed to visually evaluate data normality by comparing two probability distributions of theoretical and sample quantiles. Most data points lay close to a linear diagonal line with some points presented within the 95% confidence interval (the grey color band). [file 12871_2020_1008_MOESM3_ESM.zip › suppl figure 1.pdf]

## Slide 1
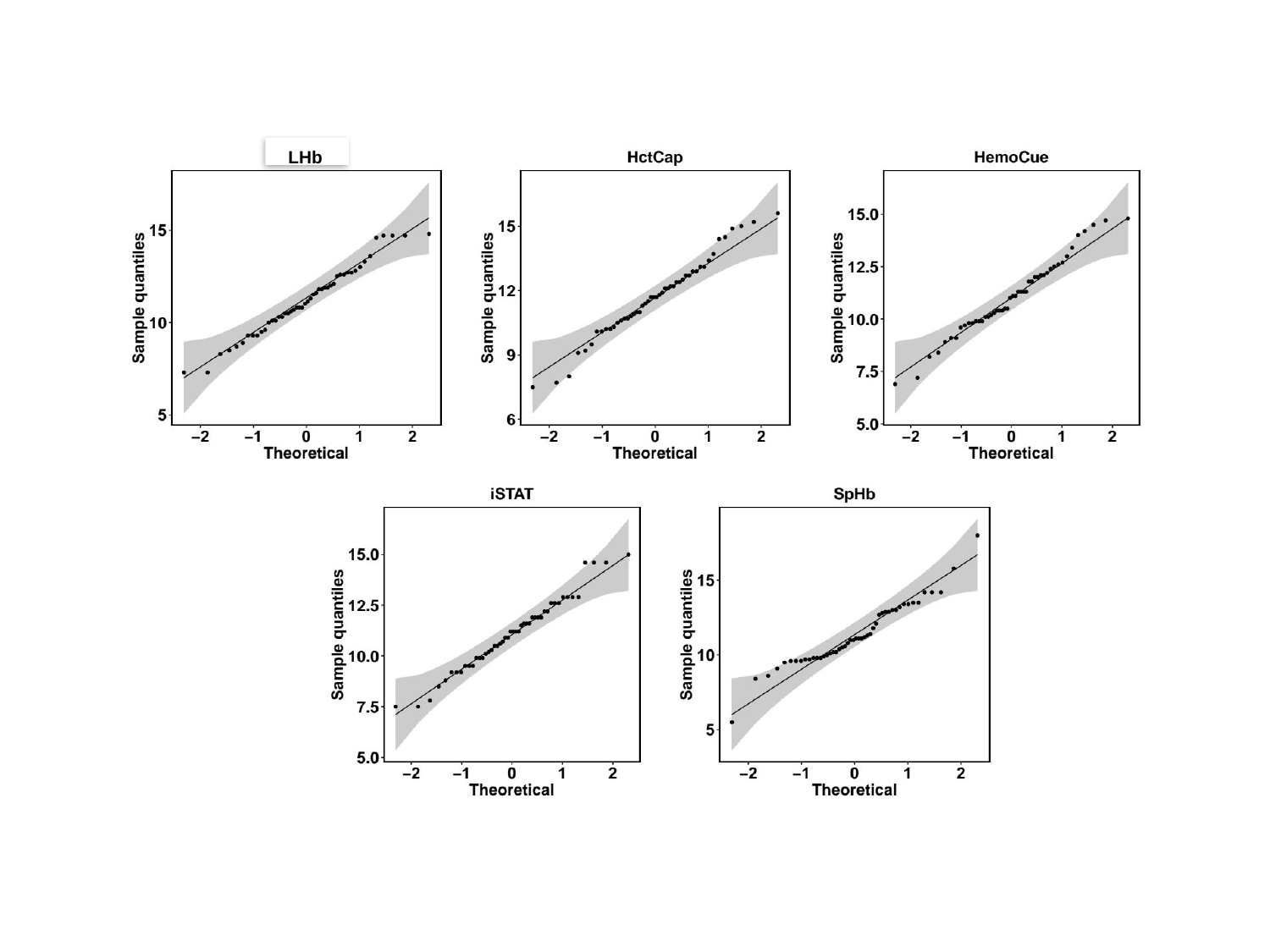

LHb

Supplement: Supplementary file 3 — Additional file 3: Figure S1. Quantile-Quantile (Q-Q) plot was performed to visually evaluate data normality by comparing two probability distributions of theoretical and sample quantiles. Most data points lay close to a linear diagonal line with some points presented within the 95% confidence interval (the grey color band). [file 12871_2020_1008_MOESM3_ESM.zip › suppl figure 1.pptx]

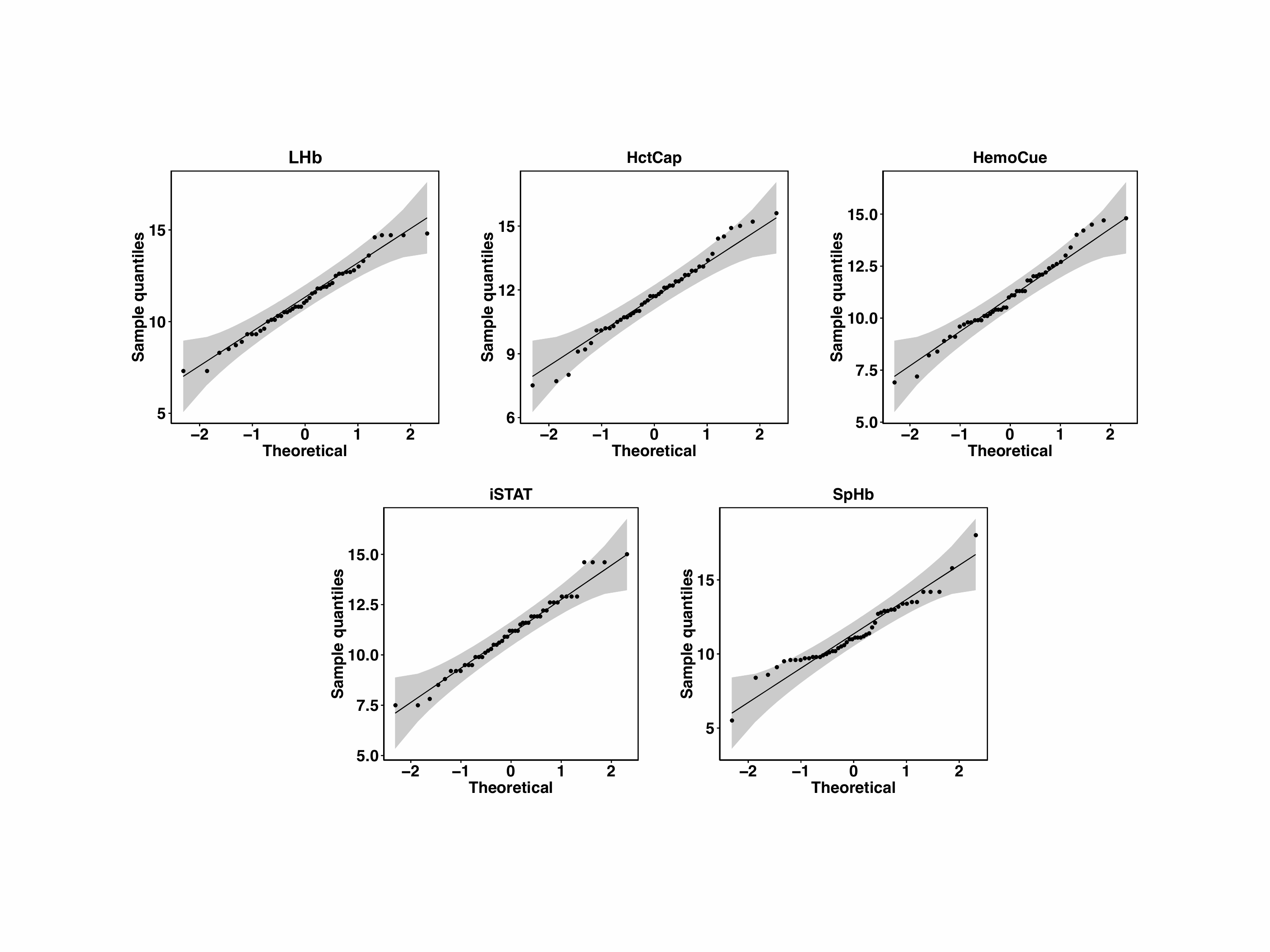

Supplement: Supplementary file 3 — Additional file 3: Figure S1. Quantile-Quantile (Q-Q) plot was performed to visually evaluate data normality by comparing two probability distributions of theoretical and sample quantiles. Most data points lay close to a linear diagonal line with some points presented within the 95% confidence interval (the grey color band). [file 12871_2020_1008_MOESM3_ESM.zip › suppl figure 1.tif]
